# Comparison between the efficacy and side effects of intravitreal versus anterior chamber Bevacizumab injection in neovascular glaucoma patients

**Published:** 2014

**Authors:** E Ungureanu, A Geamanu, V Popescu, I Dinu, M Grecescu, S Gradinaru

**Affiliations:** * ”Carol Davila” University of Medicine and Pharmacy, Bucharest, Romania; ** Department of Ophtalmolgy, University Emergency Hospital Bucharest, Romania

**Keywords:** neovascular glaucoma, Bevacizumab, anterior chamber, intravitreal

## Abstract

**Rationale.** Neovascular glaucoma is the type of glaucoma most refractory to treatment. The most frequent causes are those associated with retinal hypoxia, which promotes the upregulation of the VEGF synthesis and produces fibrovascular membranes over the anterior chamber angle.

Because the administration of anti VEGF products is still off label for neovascular glaucoma, there is not a single accepted way of treatment. There are differences between the site of administration (vitreal or anterior chamber or both at the same time), the dose or the setting of the procedure.

**Objective.** The objective of our study was to asses the difference of efficacy and complications of bevacizumab injection for vitreal administration versus anterior chamber administration.

**Methods and results.** Prospective study with 18 eyes from 18 patients with neovascular glaucoma associated with proliferative diabetic retinopathy or retinal vein occlusion. Group A (10 patients) received intravitreal injection with 0.05 ml Bevacizumab. Group B (8 patients) received anterior chamber injection with 0.03 ml Bevacizumab.

There was a significant decrease of intraocular pressure (p<0.01 for group A, p<0.05 for group B) for both groups. Group A also had a statistically significant decrease of the macular edema (p<0.05). The side effects were reduced for both groups.

**Discussion.** Our conclusion was that for the neovascular glaucoma, which associates significant macular edema, the intravitreal procedure should be performed, while for neovascular glaucoma patients without retinal edema, the anterior chamber procedure should be preferred because of reduced potential side effects.

## Introduction

Neovascular glaucoma is a secondary type of glaucoma that was first described in 1871. It is determined by the development of a fibrovascular membrane over the pupillary border, the anterior surface of the iris and especially over the anterior chamber angle.

The most frequent causes are those associated with retinal hypoxia, such as proliferative diabetic retinopathy, central retinal vein occlusion, branch retinal vein occlusion, central retinal arterial occlusion, ischemic ocular syndrome, etc.

Retinal hypoxia promotes the upregulation of the VEGF synthesis that determines the apparition and development of new vessels not only for the retina, but also on the anterior surface of the iris (rubeosis iridis) and on the anterior chamber angle. These vessels are associated with fibrotic tissue proliferation, hence fibrovascular membranes develop over the anterior chamber angle and determine neovascular glaucoma.

VEGF inhibitors have been proved as efficient for neovascular glaucoma patients. For both the more common intravitreal administration [**1**,**2**] and the anterior chamber administration [**3**], they determine a spectacular remission of the neovascularization, usually achieved in 24-48 hours [**4**,**5**]. The efficacy is maintained for 1-3 months. If the patient has a secondary open angle neovascular glaucoma, it is possible that a single bevacizumab injection, without any other treatment is enough to achieve intraocular pressure targeting for the efficacy period. For patients with secondary closed angle neovascular glaucoma and patients with secondary open angle neovascular glaucoma for whom the injection was not able to achieve the target pressure, the bevacizumab injection is still useful to determine temporary fibrovascular membrane remission, associated or not with the decrease of macular edema, and to allow a window of opportunity for other procedures, such as surgery or panretinal photocoagulation [**6**].

The objective of our study was to asses the difference of efficacy and complications of bevacizumab injection for vitreal administration versus anterior chamber administration.

## Methods

This was a prospective study with 18 consecutive eyes from 18 patients with neovascular glaucoma associated with proliferative diabetic retinopathy or retinal vein occlusion. Patients were followed in the Ophthalmology Clinic of Bucharest University Hospital between March 2013 and February 2014. 9 patients with central retinal vein occlusion, 4 patients with branch retinal vein occlusion and 5 patients with proliferative diabetic retinopathy were found. The patients have not been previously undergone glaucoma surgery, anti-VEGF injections or corticosteroid injections. 4 of the diabetic retinopathy patients have undergone previous retinal photocoagulation. 15 patients were under treatment with fixed timolol-dorzolamide combination while 3 received only timolol treatment. 

Slit lamp examination of the anterior pole and retina was performed first for all the patients. The presence of the fibrovascular membrane over the angle was assessed through gonioscopy. When possible, OCT assessed the presence of macular edema. The intraocular pressure was measured with the Goldman tonometer.

Group A consisted of 10 patients (7 with central retinal vein occlusion, 1 with branch retinal vein occlusion and 2 with proliferative diabetic retinopathy) who received intravitreal injection with 0.05 ml Bevacizumab through a 30-gauge needle. Before the procedure, the mean intraocular pressure was 27.8 mm Hg (25-36 mm Hg). 6 patients also presented macular edema (for those, mean central width was of 377 microns).

Group B consisted of 8 patients (2 with central retinal vein occlusion, 3 with branch retinal vein occlusion and 3 with proliferative diabetic retinopathy) who received anterior chamber injection with 0.03 ml Bevacizumab through a 30 gauge needle. Before the procedure, the mean intraocular pressure was 26.8 mm Hg (24-30 mm Hg). 3 patients also presented macular edema, with mean central width of 352 microns.

All the procedures were performed in the operating room theater under sterile conditions. Patients were checked the 1st day, then after 1 week, after 3 weeks and after 8 weeks following the procedure. Assessments of the presence of fibrovascular membranes of the angle, of rubeosis iridis, of retinal edema and of possible complications were performed.

## Results

The results for both groups were good with temporary resolution of the fibrovascular membranes.

For group A (**Table 1**), at 1-week reevaluation, all the patients achieved an apparent resorption of the iris and the angle fibrovascular membranes observed through slit-lamp examinations and gonioscopy. Though that, it was known that the angle membranes are sometimes difficult to observe through gonioscopy. The intraocular pressure had significantly decreased, with a mean pressure of 22.4 mm Hg (p<0.01). For the 6 patients with macular edema, there was also a significant reduction of the edema (mean of 302 microns, p<0.05). For 3 patients, the target pressure of 22 mm Hg under the previous topical treatment was not achieved and they were scheduled for trabeculectomy. After 3 weeks, there were no significant modifications. After 8 weeks, 1 patient presented repermeabilization of some iris ghost vessels.

**Table 1:** Group A at 1-week reevaluation

	Cause of neovascular glaucoma	Intraocular pressure before procedure (in mm Hg)	Intraocular pressure after procedure (at 1 week) (in mm Hg)
1	Central retinal vein occlusion	30	29
2	Central retinal vein occlusion	27	21
3	Diabetic retinopathy	28	22
4	Branch retinal vein occlusion	25	17
5	Central retinal vein occlusion	26	22
6	Central retinal vein occlusion	28	19
7	Central retinal vein occlusion	27	25
8	Diabetic retinopathy	26	21
9	Central retinal vein occlusion	25	20
10	Central retinal vein occlusion	36	28

For group B, at 1-week evaluation, all the patients achieved an apparent resorption of fibrovascular membranes at slit-lamp and gonioscopy examination. The intraocular pressure had also significantly decreased (mean pressure 23.1 mm Hg, p <0.05). Although there was a mean reduction of the macular edema (336 microns), this was not statistically significant (p=0.23). For 3 patients, the target pressure of 22 mm Hg under previous topical treatment was not achieved. They were scheduled for trabeculectomy. For the rest of the patients, there were no significant modifications at 3 weeks. After 8 weeks, 2 patients presented partial repermeabilization of the ghost vessels.

Both procedures were also safe. For Group A, 4 patients presented subconjunctival hemorrhages at the injection site, for 1 of those patients, the hemorrhage was extended at the supero-temporal quadrant of the conjunctiva.

For group B, one patient presented hyphema, which had disappeared at the 1 week reevaluation.

**Table 2:** Group B at 1-week evaluation

	Cause of neovascular glaucoma	Intraocular pressure before procedure (in mm Hg)	Intraocular pressure after procedure (at 1 week) (in mm Hg)
1	Diabetic retinopathy	29	22
2	Central retinal vein occlusion	28	26
3	Branch retinal vein occlusion	24	21
4	Branch retinal vein occlusion	25	18
5	Diabetic retinopathy	26	21
6	Diabetic retinopathy	25	22
7	Central retinal vein occlusion	28	27
8	Branch retinal vein occlusion	30	28

## Discussion

Neovascular glaucoma is the type of glaucoma most refractory to treatment. The use of anti VEGF treatment, initially used for choroidal neovascularization of wet AMD, although is still off-label for neovascular glaucoma, has significantly improved our possibilities of therapy.

Because the administration of anti VEGF products is still off label for neovascular glaucoma, there is not a single accepted way of treatment. There are differences between the site of administration (vitreal or anterior chamber or both at the same time [**5**]), the dose or the setting of the procedure (outpatient office setting or operating room).

While the site of the ischemia is the retina (which determines the production of VEGF), for neovascular glaucoma, the site of VEGF action is the anterior chamber, so there is a rationale behind both sites of administration.

Our study has tried to determine if there is an administration mode which is to be preferred. The results were good for both the vitreal and the anterior chamber administration, with the resorption of fibrovascular membranes and decrease and intraocular pressure. Unlike intravitreal administration, for anterior chamber administration, no statistically significant decrease of macular edema was achieved, although there was a try to point that the number of the patients in that group was small (8 total patients, only 3 with macular edema) and larger groups might have demonstrated a statistically significant reduction of the edema.

The side effects were reduced for this study. It is known from larger studies, that intravitreal administration has risks such as central retinal arterial occlusion [**7**] or retinal detachment, which, although very rare, are serious, with potential loss of vision. Anterior chamber administration in neovascular glaucoma may present a higher proportion of cases with hyphema.

Our conclusion was that for the neovascular glaucoma, which associated significant macular edema, the intravitreal procedure should be performed, while for the neovascular glaucoma, patients without retinal edema, the anterior chamber procedure should be preferred because of reduced possible side effects. 

**Sources of Funding**

None

**Disclosures**

None
